# The appendix as an unexpected culprit of small bowel obstruction: A case report

**DOI:** 10.1016/j.ijscr.2025.110981

**Published:** 2025-01-29

**Authors:** Daneal Weldegiorgies, Suleiman Ayalew Belay, Michael A. Negussie, Dagnachew Akalu, Eshete Endalew, Bereket Tusa

**Affiliations:** a Department of Surgery, College of Medicine and Health Sciences, University of Gondar, Gondar, Ethiopia; b School of Medicine, College of Medicine and Health Sciences, University of Gondar, Gondar, Ethiopia; c School of Medicine, College of Health Sciences, Addis Ababa University, Addis Ababa, Ethiopia

**Keywords:** Appendicular knot, Small bowel obstruction, Appendicitis, Acute abdomen, Case report

## Abstract

**Introduction:**

Appendico-ileal knotting is an extremely rare complication of acute appendicitis resulting in small bowel obstruction (SBO) and potential strangulation. It accounts for a negligible fraction of SBO cases, with only a handful of cases reported globally in the literature. The condition arises when an inflamed or gangrenous appendix forms a knot around the ileum, often exacerbated by adhesions or anatomical variations, leading to closed-loop obstruction and, if untreated, bowel strangulation and gangrene.

**Case presentation:**

A 34-year-old female presented with four days of crampy mid-abdominal pain, nausea, bilious vomiting, and absence of bowel movements. Examination revealed generalized peritonitis, diffuse tenderness with guarding, and rigidity, accompanied by leukocytosis. An emergency laparotomy uncovered hemorrhagic fluid and an inflamed appendix wrapping around the distal ileum, causing a closed-loop obstruction with necrotic-appearing bowel. Following hemicolectomy, ileostomy, and appendectomy, the patient stabilized, was weaned off vasopressors, and recovered uneventfully. Discharged on postoperative day seven and followed up regularly, she eventually underwent ileostomy reversal three months later without complications.

**Discussion:**

Acute appendicitis rarely leads to SBO through appendico-ileal knotting. Preoperative diagnosis is challenging due to its rarity and non-specific radiologic features. Surgical management varies from simple appendectomy to resection of gangrenous bowel, with decisions guided by intraoperative findings.

**Conclusion:**

High clinical suspicion, prompt recognition, and timely surgical intervention ensure better outcomes in appendico-ileal knotting.

## Highlights

• Appendico-ileal knotting is a rare cause of small bowel obstruction.
• Early surgical intervention is crucial for preventing bowel gangrene.
• Preoperative diagnosis is difficult due to non-specific imaging findings.
• Surgical management depends on intraoperative findings and bowel viability.
• Prompt treatment leads to favorable patient outcomes.

## 1 Introduction

Small bowel obstruction (SBO) is one of the most common causes of acute abdomen, often requiring surgical intervention. Appendico-ileal knotting is a rare, complicated form of acute appendicitis, which results in small bowel obstruction [1]. The appendicular knot, also known as appendicular band syndrome or appendicular tie syndrome, is an extremely rare surgical condition, with only a handful of cases reported globally in the literature [[2], [3], [4]]. It typically presents as intestinal obstruction, where the ileum becomes entrapped by the appendicular knot, leading to closed-loop obstruction. If not treated promptly, this may result in strangulation and small bowel gangrene [5]. Here, we report the case of a 34-year-old female patient who presented with SBO secondary to appendico-ileal knotting, diagnosed intraoperatively.

This case has been reported in line with the SCARE criteria [6].

## 2 Clinical presentation

A 34-year-old female from northern Ethiopia presented to the emergency surgical unit with a four-day history of progressive, crampy mid-abdominal pain that later became diffuse, predominantly in the lower abdomen. Her symptoms were accompanied by nausea, bilious vomiting, and obstipation for two days. She reported no prior abdominal surgery, trauma, medical comorbidities, or relevant family or drug history. The patient denied pelvic pain, dysmenorrhea, or symptoms suggestive of ovarian pathology, effectively ruling out gynecological causes such as cyst torsion.

On examination, she exhibited tachycardia (144 bpm), tachypnea (48 breaths/min), and a blood pressure of 130/70 mmHg, with diffuse abdominal tenderness, guarding, and rigidity. Neurologic assessment revealed a Glasgow Coma Scale (GCS) of 14/15 (E4V4M6) contributing to a Quick Sequential Organ Failure Assessment (qSOFA) score of 2, consistent with early sepsis. Laboratory findings included leukocytosis (19,300/μL, 88 % neutrophils) and a hematocrit of 38 %. Due to hemodynamic instability and clinical signs of generalized peritonitis, imaging was deferred, and she was taken urgently for exploratory laparotomy. Preoperative management included aggressive crystalloid resuscitation and empiric broad-spectrum antibiotics.

**Fig. 1:**
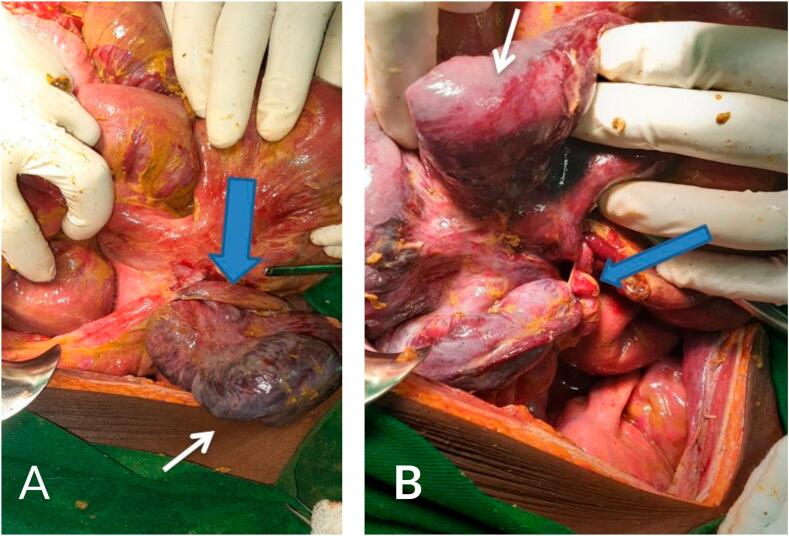
(A and B): (A) Intraoperative image demonstrating the inflamed appendix (blue arrows) wrapping tightly around a segment of distal ileum, forming a closed-loop obstruction near the ileocecal valve. The appendix tip adheres to the cecum, contributing to a tourniquet-like effect. (B) A closer view of the same intraoperative findings, highlighting the ischemic and necrotic appearance of the affected distal small bowel segment (white arrows) caused by the knot formed by the appendix, which impaired vascular flow to the segment.

Intraoperatively, 1.5 l of hemorrhagic peritoneal fluid was evacuated, and an inflamed appendix was found wrapped around a 60 cm segment of distal ileum near the ileocecal valve, forming a closed-loop obstruction (Fig. 1). The appendix tip adhered to the cecum, creating a tourniquet effect that resulted in a segment of ischemic, necrotic-appearing ileum without evidence of perforation. Persistent intraoperative hypotension (80/50 mmHg) and tachycardia (130–140 bpm) necessitated norepinephrine infusion. The affected ileum was resected, and a right hemicolectomy was performed. An end ileostomy was created due to the patient's hemodynamic instability and the associated high risk of anastomotic leak. Histopathological evaluation was deferred due to financial constraints.

Postoperatively, the patient was transferred to the surgical ICU. Vasopressor support was discontinued after 24 h, and following a three-day stay in the ICU, she was moved to the surgical ward. The patient experienced a smooth postoperative course with stable hemodynamics, adequate pain control, and satisfactory wound healing. She was discharged on the seventh postoperative day with plans for follow-up regarding ileostomy reversal and monitoring of her recovery.

The patient was followed up in the surgical outpatient department, and three months later, an ileostomy reversal was performed. She had an uneventful postoperative period and was discharged home in good condition.

## 3 Discussion

Multiple etiologies have been reported for small bowel obstruction. Acute appendicitis is a very rare cause of small bowel obstruction. The first such case was reported by Hotchkiss in 1901 [7]. In 1909, Hawks [8] divided the causes into mechanical and septic appendicitis (adynamic), or a combination of both. Small bowel obstruction as a complication of acute appendicitis may be either dynamic or mechanical. Paralytic ileus caused by appendicular inflammation is the most common cause of intestinal obstruction in acute appendicitis, occurring in 1–5 % of cases [1].

Strangulation of the obstructed bowel may result from a long-standing closed-loop obstruction, which can be due to a chronically inflamed appendix constricting around a loop of the small bowel, as in our case, or when the appendix is adhered to surrounding structures and a portion of the bowel herniates through the resulting gap. Among the mechanical causes, the vast majority result from the formation of an appendicular abscess that compresses loops of the small bowel, or from postoperative adhesions [9]. There are two basic situations in which the appendix may cause mechanical obstruction [10]: an appendicular tip attached to the mesentery surrounding an ileal loop, compressing its lumen; or an appendicular tip attached to the intestinal serosa, causing obstruction by direct compression or torsion of the loop.

Fekadu et al., in a systematic review conducted in Ethiopia, identified small bowel volvulus as the most common cause of SBO, followed by intussusception and adhesion. Other significant causes included hernias and ileo-sigmoid knotting, while rare causes such as ascariasis, gossypiboma, and gallstone ileus were also reported [11].

Preoperative diagnosis of appendico-ileal knotting as a secondary cause of SBO is quite challenging. Appendico-ileal knotting does not have more specific features than other causes of SBO. The rarity of these cases, combined with limited clinical experience, often leads to misdiagnosis. Though the radiologist's experience plays a vital role, the scarcity of imaging modalities such as abdominal CT scans [[12], [13], [14]] limits the ability to diagnose this condition preoperatively, resulting in confirmation only during surgery. Management options are determined by intraoperative findings.

Our patient presented four days after the onset of illness with signs of generalized peritonitis and shock, leading us to consider gangrenous small bowel volvulus. The management of appendico-ileal knotting mainly depends on the bowel segment involved and the level of strangulation. Surgical treatment ranges from appendectomy to resection of any gangrenous bowel [15]. Intraoperatively, if the bowel is viable, simply untying the knot may suffice. However, if the bowel is ischemic, non-viable, or gangrenous, resection of all non-viable segments with subsequent anastomosis is required, as in our case.

## 4 Conclusion

Appendico-ileal knotting is a very rare cause of intestinal obstruction. A high index of suspicion is crucial for diagnosis, as delays may significantly increase the risk of morbidity and mortality. Early diagnosis and treatment are associated with favorable outcomes.

## Author contribution

**Daneal Weldegiorgies**: Writing – original draft, Conceptualization, Resources. **Suleiman Ayalew Belay**: Writing – original draft, Conceptualization, Resources. **Michael A. Negussie**: Writing – review & editing, Visualization. **Dagnachew Akalu**: Writing – review & editing, Data curation. **Eshete Endalew**: Writing – review & editing, Resources. **Bereket Tusa**: Supervision.

## Consent for publication

Written informed consent was obtained from the patient for publication of this case report and accompanying images. A copy of the written consent is available for review by the Editor-in-Chief of this journal on request.

## Ethical approval

Ethical approval for this case report was provided by the Department of Surgery, College of Medicine and Health Sciences, University of Gondar, Gondar, Ethiopia (ethical approval number was not provided).

## Guarantor

Dr. Daneal Weldegiorgies.

## Research registration number

N/A.

## Funding

No source of funding is provided for this case report.

## Conflict of interest statement

The authors declare that they have no known competing financial interests or personal relationships that could have appeared to influence the work reported in this paper.
